# Systematic review of general burden of disease studies using disability-adjusted life years

**DOI:** 10.1186/1478-7954-10-21

**Published:** 2012-11-01

**Authors:** Suzanne Polinder, Juanita A Haagsma, Claudia Stein, Arie H Havelaar

**Affiliations:** 1 Department of Public Health, Erasmus MC, Rotterdam, Netherlands; 2 Division of Information, Evidence, Research and Innovation, WHO Regional Office for Europe, Copenhagen, Denmark; 3 National Institute for Public Health and the Environment, Laboratory for Zoonoses and Environmental Microbiology, Bilthoven, Netherlands; 4 University Utrecht, Institute for Risk Assessment Sciences, Utrecht, Netherlands

**Keywords:** Review, General burden of disease, Disability-adjusted life years, Methodology

## Abstract

**Objective:**

To systematically review the methodology of general burden of disease studies. Three key questions were addressed: 1) what was the quality of the data, 2) which methodological choices were made to calculate disability adjusted life years (DALYs), and 3) were uncertainty and risk factor analyses performed? Furthermore, DALY outcomes of the included studies were compared.

**Methods:**

Burden of disease studies (1990 to 2011) in international peer-reviewed journals and in grey literature were identified with main inclusion criteria being multiple-cause studies that quantified the burden of disease as the sum of the burden of all distinct diseases expressed in DALYs. Electronic database searches included Medline (PubMed), EMBASE, and Web of Science. Studies were collated by study population, design, methods used to measure mortality and morbidity, risk factor analyses, and evaluation of results.

**Results:**

Thirty-one studies met the inclusion criteria of our review. Overall, studies followed the Global Burden of Disease (GBD) approach. However, considerable variation existed in disability weights, discounting, age-weighting, and adjustments for uncertainty. Few studies reported whether mortality data were corrected for missing data or underreporting. Comparison with the GBD DALY outcomes by country revealed that for some studies DALY estimates were of similar magnitude; others reported DALY estimates that were two times higher or lower.

**Conclusions:**

Overcoming “error” variation due to the use of different methodologies and low-quality data is a critical priority for advancing burden of disease studies. This can enlarge the detection of true variation in DALY outcomes between populations or over time.

## Introduction

The burden of disease concept provides a conceptual and methodological framework to quantify and compare the health of populations using a summary measure of both mortality and disability – the disability-adjusted life year (DALY) [1,2]. Since the launch of the Global Burden of Disease (GBD) study in 1993, the burden of disease concept has been widely adopted by countries and health development agencies alike to identify the relative magnitude of different health problems. This information serves as crucial input for debates about priorities in the health sector.

Criticism of the GBD study focused on the construction of DALYs [3,4], particularly the social choices around age weights and severity scores of disabilities. The GBD 2010 Study that is currently being conducted responded to the critiques and recent improvements in the field and includes significantly improved methods for burden assessment, particularly for ranking risk factors and disabilities [5,6]. It is expected that the imminent publication of the GBD 2010 Study will result in a new impulse to perform burden of disease studies.

A major strength of the burden of disease concept is that it allows comparison between different health problems, between different years, and between countries. In principle, the DALY approach should be used consistently to provide comparable DALY estimates. However, the technical approach of the GBD is complex, both in concept and in application, and there are many methodological alternatives, e.g., using alternative morbidity estimates, life expectancies, or severity weights, which have enormous influence on DALY outcomes [7]. Hence, the interpretation of results of burden of disease studies requires detailed methodological knowledge.

General burden of disease studies are multiple-cause studies that quantify the burden of disease as the sum of the burden of all distinct diseases expressed in DALYs. Until now, a systematic review of general burden of disease studies and the underlying methodological choices has not been conducted. This review was a first step in the development of a protocol specifically for burden of foodborne disease studies. This protocol complements the GBD manual, as it addresses problems that arise particularly when undertaking foodborne burden of disease studies. The protocol was developed for researchers that aim to undertake burden of foodborne disease studies in the framework of the Foodborne Disease Burden Epidemiology Reference Group (FERG). The FERG was established in 2007 by the World Health Organization (WHO). The purpose of the group is to advise WHO in their estimates of the global burden of diseases commonly transmitted through food.

This systematic review aims to provide an overview of the methodology of general burden of disease studies using the DALY approach. In the review, the following key questions were addressed: 1) what was the quality of the data and were there any data gaps, 2) which methodological choices were made in order to calculate years of life lost due to mortality (YLL) and years lost due to disability (YLD), and 3) which methods were used to handle uncertainty and risk factor analysis. Furthermore, DALY outcomes for specific disease and injury groups resulting from the general burden of disease studies were compared.

## Methods

### Selection criteria

In this review, burden of disease studies based on general multiple-cause studies (including all diseases and injuries) were included. Empirical studies in international peer-reviewed journals and grey literature published in English in the period 1990 to 2011 were included. Studies in established market economies and low- and middle-income countries were also included. The review is restricted to studies using the DALY as a burden of disease measure, both country-specific and worldwide.

### Disability-adjusted life year

**Figure 1:**
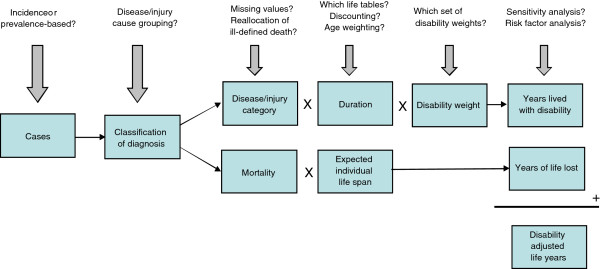
Conceptual model.

The DALY is calculated by adding YLL to morbidity and disability, expressed in YLD. The DALY methodology is represented in a conceptual framework in Figure 1.

YLL is calculated by summation of the number of fatal cases (d) due to health outcome (x) in a certain period multiplied by the residual expected life expectancy (e) at the age of death:

$${\text{YLL}}_{\text{x}}=\Sigma d_{x}\times e_{x}$$

For the calculation of YLD an incidence or prevalence-based approach can be used, which is highly dependent on the availability of data. The incidence-based approach quantifies both the burden of disease occurring during the reference period and the burden accrued into the future. A prevalence-based approach ascribes burden to the age at which disability is lived.

YLD_inc_ is calculated by multiplying the number of incident cases (I) at a certain age with health outcome (x) by the duration of the health outcome (t) and the disability weight (dw) assigned to health outcome x:

$${\text{YLD}}_{\text{incx}}=\Sigma I_{x}\times t_{x}\times {\mathrm{dw}}_{x}$$

YLD_prev_ is calculated by multiplying the number of prevalent cases (P) in age group (x) at a point in the reference period with the disability weight (dw) assigned to health outcome x:YLD_prev x_ = P_x_ × dw_x_ These basic formulas can be supplemented due to methodological choices (e.g., expanding with discount factor and age-weighting).

### Data sources and search strategy

Searches of eligible studies were conducted in Medline (PubMed), EMBASE, and Web of Science. Searches for eligible grey literature were conducted in Google Scholar and SIGLE (System for Information on Grey Literature in Europe). All international peer-reviewed articles and grey literature published in English in the period January 1990 to 2011 were included in the searches. Search terms used for general burden of disease studies were: “burden of disease, “disability adjusted life year,” “disability-adjusted life year,” “DALY.” Keywords were matched to database-specific indexing terms. In addition to database searches, reference lists of review studies and articles included in the review were screened for titles that included key terms.

### Data extraction

Relevant papers were selected by screening the titles (first step), abstracts (second step), and entire articles (third step) retrieved through the database searches. During each step respectively, the title, abstract, or entire article was screened to ensure that it met the selection criteria listed above. This screening was conducted independently by two researchers (Suzanne Polinder and Juanita Haagsma). Disagreement about eligibility between the reviewers was solved through discussion.

Full articles were critically appraised by two reviewers (Suzanne Polinder and Juanita Haagsma), using data extraction forms, which included information on the study population, details regarding the methods used to calculate YLL and YLD, risk factor analysis, main conclusions, and evaluation of results. Their reports were compared and disagreements were resolved by discussion.

## Results

### Literature search

**Figure 2:**
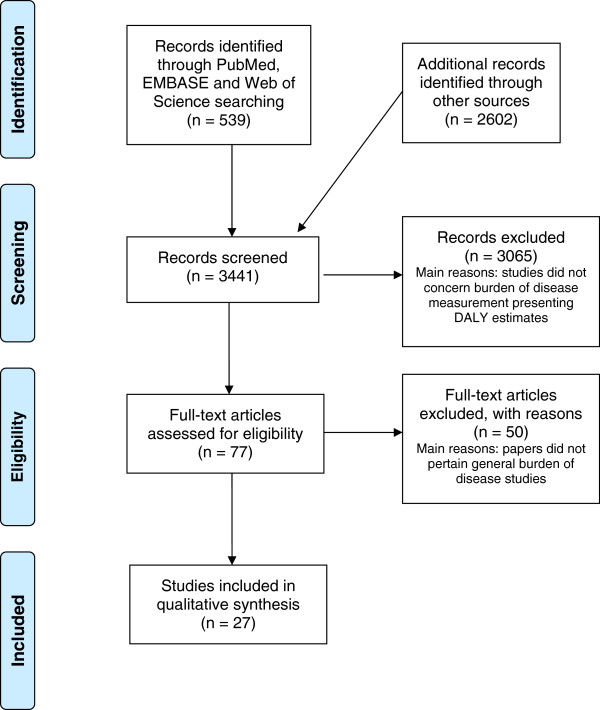
Flow diagram of the search of existing burden of disease studies.

**Figure 3:**
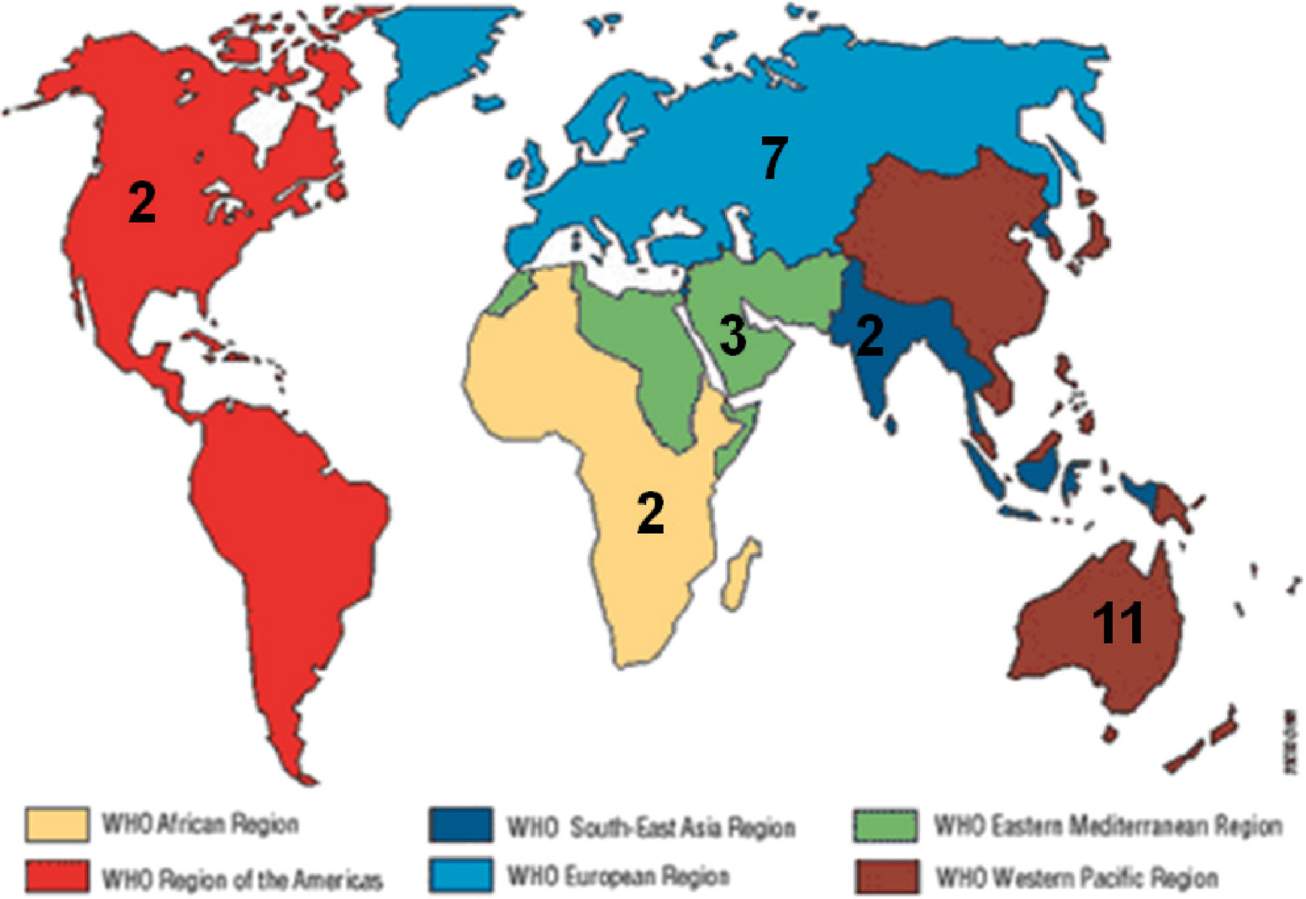
Number of general burden of disease studies, per WHO region.

Figure 2 shows the flow diagram of the search of existing burden of disease studies and main reasons for exclusion. Eventually, 31 studies were included in the review. Table 1 shows the studies that have been included for the review. In Figure 3, the number of general burden of disease studies is shown per WHO region. Four studies were worldwide burden of disease studies [8-11].

**Table 1:** Study characteristics of included studies

**Author, Year (reference no.)**	**Country**^**1**^	**WHO region**	**Data sources mortality**	**Data sources morbidity**
Begg, 2008 [13]	Australia	WPRO	Australian death registry from Australian Bureau of Statistics	Disease registers, cohort and intervention studies and surveys
Bowie, 1997 [22]	South and West region, England	EURO	1992 Office of Population Censuses and Surveys age-specific mortality data	World bank study
Bradshaw, 2003 [14]	South Africa	AFRO	1) Statistics South Africa, 2) Population Register, 3) National Injury Mortality Surveillance Study, 4) For children: 1996 census and the 1998 Demographic and Health Survey	The South African Birth Defects Surveillance System
Bundhamchareon, 2002 [16]	Thailand	SEARO	Survey of Population Change	Disease registers, routine databases or epidemiological studies
Chapman, 2006 [41]	Zimbabwe	AFRO	Vital Registration System and number of deaths for 1997 taken from a nationwide census	Local disease registers, surveys and routine health service data supplemented by estimates from epidemiological studies from other settings
Dodhia, 2008 [50]	London, England	EURO	Local mortality data	The GBD estimates from World Health Organization were used
Hyder, 2000 [40]	Pakistan	EMRO	Pakistan Demographic Survey of 1989, Pakistan Demographic and Health Survey 1990–1991, National Health Survey of Pakistan 1989–1994	More than 180 national and local data sources were reviewed to obtain information on diseases in Pakistan
Innove Solutions, 1998 [51]	West Pennine, England	EURO	National mortality data from Public Health Department	West Pennine Health Authority morbidity data
Jankovic, 2007 [52]	Serbia and Serbia Montenegro	EURO	Serbian Office of Statistics mortality database	Disease registers, routine databases, and epidemiological studies
Laaser, 2007 [38]	Syria	EMRO	National mortality register, analysis of the WHO life table of Syria and mortality sentinel surveillance	National databases
Lai, 2009 [27]	Estonia	EURO	Vital registration of Statistics Estonia	Estonian Health Insurance Fund database
Lopez, 2006 [9]	Global	Global	Death registrations, population-based epidemiological studies, disease registers, and notification systems.	Disease registers, epidemiological studies, health surveys, and health facility data
Mathers, 2001 [28]	Australia	WPRO	Australian death registry from Australian Bureau of Statistics	Disease registers and epidemiological studies
Melse, 2000 [21]	Netherlands		Dutch death registration	General practitioner registrations, national registries, and population surveys
Michaud, 2006 [35]	United States	AMRO	Mortality File from the National Center for Health Statistics 1996	National health surveys, the National Hospital Discharge Database, disease registers, and epidemiological studies
Murray, 1997 [10]	Global	21 regions in the world	Death registration systems, sample death registration systems, epidemiological assessments, cause of death models	Disease registers, population surveys, epidemiological studies, health facility data
Naghavi, 2009 [31]	Iran	EMRO	data from the national death registry of Health Ministry ME	Disease surveillance systems, hospital disease registries, representative national surveys, subnational and local studies.
Phua, 2009 [39]	Singapore, Malaysia	WPRO	Registry of Births and Deaths	National disease registers and surveillance or notification systems, national health surveys, health services utilization data
Pike, 2002 [30]	Queensland, Australia	WPRO	Australian death registry from Australian Bureau of Statistics	Australian BoD YLD data
SA Department of Health Australia, 2005 [12]	South Australia	WPRO	Australian death registry from Australian Bureau of Statistics	Disease registers and epidemiological studies
Somerford, 2004 [29]	Western Australia	WPRO	Australian death registry from Australian Bureau of Statistics	Australian BoD YLD data
Stevens, 2008 [26]	Mexico	AMRO	National Mortality statistics	Vital statistics, national censuses, health examination surveys, and published epidemiological studies
Tobias, 2001 [20]	New Zealand	WPRO	New Zealand Health Information Service mortality	Disease registers, population surveys, hospital discharge register
Ünüvar, 2004 [15]	Turkey	EURO	Death statistics obtained from provincial and town centers in Turkey, hospital records and Directorate General of Security data	Population Census, Records obtained from government agencies, National surveys, national and international reports and articles
Victorian BOD, 2005 [19]	Victoria, Australia	WPRO	Australian death registry from Australian Bureau of Statistics	Disease registers, routine databases and population health surveys
World Health Organization, 2008 [8]	Global	21 regions in the world	Death registration systems, sample death registration systems, epidemiological assessments, cause of death models	Disease registers, population surveys, epidemiological studies, health facility data
World Health Organization, 2009 [11]	Global	21 regions in the world	Death registration systems, sample death registration systems, epidemiological assessments, cause of death models	Disease registers, population surveys, epidemiological studies, health facility data
Yoon, 2007 [24]	Korea	SEARO	The Korean National Health Insurance system	Large normative cohort
Yusoff, 2004 [17]	Malaysia	WPRO	Malaysian mortality data of the Department of Statistics subdivided in four regions	Disease registries, routine databases and epidemiological studies
Zhao, 2004 [18]	Northern Territory, Australia	WPRO	Australian death registry from Australian Bureau of Statistics	Disease registers, population surveys, expert opinions
Zhou, 2011 [36]	Yunnan Province, China	WPRO	Medical death certificate information from Centers for Disease Control and Prevention	Indirect method: YLD/YLL ratio for China taken from the WHO World Health Report 2002

^1^ Country / region and country of the study population.

### What was the quality of the data and were there any data gaps?

The availability and quality of mortality and morbidity data strongly differs by country.

Most countries register the number of fatal cases as well as the age and cause of death in national vital registrations (see Table 1). Vital registration often had full coverage, which means that the data are representative for the population of these countries.

Where vital registration had less than 100% coverage it is important to know whether the data had been extrapolated to 100%. However, the majority of the studies did not report whether the death statistics used had 100% coverage. Only 10 studies reported that they corrected for underreporting of death statistics [8,10-18], for instance with demographic projection models [14], or by using the average of several years of death statistics to minimize stochastic variation [10,18].

Next to extrapolation in case of missing data, procedures such as reallocating of ill-defined deaths from so-called “garbage codes” may be necessary. Problems can arise from the routine use of specific codes in the International Classification of Diseases (ICD) list where information is incomplete. This can occur when medical records are not fully considered or when medical practitioners concerned with the specific cases are not consulted during the process of completing the forms. Certain codes then become overused and bias the relative importance attached to particular cases of death. These codes are called “garbage codes.” The majority of the studies (n=24; Table 2) reported corrections for ill-defined deaths, partly by reallocation from garbage codes. For instance, the Victorian Burden of Disease study [19] redistributed the cardiovascular garbage codes to ischemic heart disease, inflammatory heart disease, and hypertensive heart disease in proportions that varied by age. Notably, many studies did not report whether mortality data were corrected for missing data, underreporting, or misclassification.

**Table 2:** Methods used to calculate YLL and YLD in general burden of disease studies

**Author, Year (reference no.)**	**General burden of disease methods**							**YLL methodology**		**YLD methodology**		
	**Incidence or prevalence based approach**	**Disease and injury cause groups used**	**Were missing values handled?**	**Discounting**	**Age weighting**	**Sensitivity analysis**	**Risk factor analysis**	**Which life tables used?**	**Reallocation of ill-defined death? (y/n)**	**Disability weights**	**Distribution by severity?**	**Adjustment for comorbidity**
**GBD approach**	**Incidence**	**GBD list I, II, III**	**Y**	**Y**	**Y**	**Y**	**Y**	**Standard West 25 (males) and 26 (females)**	**Y**	**GBD**	**N**	**N**
Begg, 2008 [13]	Incidence	GBD list I, II, III	Y	Y	Y	Y	Y	Standard West 25 (females) and 26 (males)	Y	GBD, DDW	Y	Y
Bowie, 1997 [22]	Incidence	Own developed groups	n.a.	Y	Y	N	N	Standard West 25 (females) and 26 (males)	N	GBD	N	N
Bradshaw, 2003 [14]	Incidence	Adapted GBD list (removed causes due to little relevance)	Y	Y	Y	Y	N	Standard West 25 (males) and 26 (females)	Y	GBD1990, GBD2000, Audw, DDW	Y	N
Bundhamcharoen, 2002 [16]	Incidence	GBD list I, II, III	Y	Y	Y	Y	N	Standard West 26	Y	GBD, DDW	Y	Y
Chapman, 2006 [41]	Incidence	GBD list I, II, III	Y	Y	Y	Y	N	Standard West 26	Y	GBD, Zimb	N	n.a.
Dodhia, 2008 [50]	Incidence	Adapted GBD list (removed causes and added own groups)	Y	Y	N	Y	N	Standard West 26	N	GBD	N	N
Hyder, 2000 [40]	Incidence	Adapted GBD list (removed causes and added own groups)	n.a.	Y	N	Y	N	Standard West 25 (females) and 26 (males)	N	GBD	N	N
Innove Solutions, 1998 [51]	Incidence	Adapted GBD list (removed causes and added own groups)	N	N	N	N	N	Standard West 26	Y	GBD, DDW	N	n.a.
Jankovic, 2006 [52]	Incidence	Adapted GBD list (removed causes due to little relevance)	Y	Y	Y	Y	N	Standard West 26	n.a.	GBD1990, GBD2000, DDW	Y	N
Laaser, 2007 [38]	Incidence and prevalence	GBD list I, II, III	n.a.	Y	Y	Y	N	Standard West 25 (females) and 26 (males)	Y	GBD1996, GBD2000	n.a.	N
Lai, 2009 [27]	Prevalence	GBD list I, II, III	Y	N	N	N	N	Standard life tables of Estonia	N	Estdw	Y	N
Lopez, 2006 [9]	Incidence	GBD list I, II, III	n.a.	Y	N	Y	Y	Standard West 25 (females) and 26 (males)	Y	GBD	n.a.	N
Mathers, 2001 [28]	Incidence	Adapted GBD list (added own groups)	Y	Y	N	Y	Y	Australian standard life tables	Y	DDW, GBD	Y	Y
Melse, 2000 [21]	Prevalence	Own developed groups	n.a.	N	N	Y	N	Dutch life tables for 1994	Y	DDW	Y	N
Michaud, 2006 [35]	Incidence	Adapted GBD list (removed causes and added own groups)	Y	Y	Y	Y	N	Standard West level 25 and 26	Y	GBD	Y	N
Murray, 1997 [10]	Incidence	GBD list I, II, III	n.a.	Y	Y	Y	Y	Standard West level 25 and 26	Y	GBD	n.a.	N
Naghavil, 2009 [31]	Incidence	Adapted GBD list (removed causes and added own groups)	Y	Y	Y	N	N	Iran life expectancy	Y	GBD, DDW	n.a.	Y
Phua, 2009 [39]	Incidence	Adapted GBD list (removed causes and added own groups)	Y	Y	N	N	N	Standard West 25 (females) and 26 (males)	Y	GBD, DDW, Audw	Y	N
Pike, 2002 [30]	Incidence	Adapted GBD list (removed causes and added own groups)	n.a.	Y	N	N	N	Australian standard life tables	n.a.	GBD, DDW	Y	Y
SA Department of Health Australia, 2005 [12]	Incidence	Adapted GBD list (added own groups)	Y	Y	Y	Y	Y	Australian standard life tables	Y	GBD, DDW	Y	Y
Somerford, 2004 [29]	Incidence	GBD list I, II, III	N	Y	Y	Y	Y	Australian standard life tables	Y	N, YLL:YLD	N	N
Stevens, 2008 [26]	Incidence	GBD list I, II, III	n.a.	Y	Y	N	Y	Standard life tables of Mexico	Y	GBD	N	N
Tobias, 2001 [20]	Incidence	Own developed groups	Y	Y	Y	Y	Y	Standard West 25 (females) and 26 (males)	n.a.	DDW, GBD, Audw, EQ5Ddw	Y	Y
Ünüvar, 2006 [15]	Incidence	GBD list I, II, III	Y	Y	Y	Y	Y	Standard West	Y	GBD, DDW	Y	N
Victorian BoD study, 2005 [19]	Incidence	Adapted GBD list (removed causes and added own groups)	Y	Y	N	Y	Y	Standard West 25 (females) and 26 (males)	Y	DDW, GBD, EQ5D, Audw	Y	Y
World Health Organization, 2008 [8]	Incidence	GBD list I, II, III	Y	Y	Y	Y	Y	Standard West level 25 and 26	Y	GBD	N	N
World Health Organization, 2009 [11]	Incidence	GBD list I, II, III	Y	Y	Y	Y	Y	Standard West level 25 and 26	Y	GBD	N	N
Yoon, 2007 [24]	n.a.	Adapted GBD list (removed causes and added own groups)	Y	n.a.	n.a.	Y	N	Standard West	Y	KORdw	Y	N
Yusoff, 2004 [17]	Incidence	GBD list I, II, III	Y	Y	N	N	N	Standard West 25 (females) and 26 (males)	Y	GBD, DDW	Y	N
Zhao, 2004 [18]	Incidence	GBD list I, II, III	n.a.	n.a.	n.a.	N	N	Australian standard life tables	Y	DDW, GBD	n.a.	N
Zhou, 2011 [36]	Prevalence	Adapted GBD list (removed causes)	n.a.	Y	n.a.	N	N	Standard West	Y	N, YLL:YLD	n.a.	N

Abbreviations disability weights: Disability weights: GBD1990/GBD 1996 = Global Burden of Disease disability weights 1990 [1], GBD2000 = Global Burden of Disease disability weights 1990 supplemented with Dutch Disability weighs, DDW= Dutch Disability weights [53], AUdw = Australian NBD disability weights [25], Zimb = disability weights based on preferences of Zimbabwe [41], KORdw= Korean disability weights [24], Estdw = Estonian disability weights [27], EQ5Ddw = EQ5D disability weights [20].

### Which methodological choices were made in order to calculate YLL and YLD

#### General burden of disease methods

An incidence- or prevalence-based method can be used to quantify the burden of disease. In practice, it is often difficult to rigidly apply the incidence or prevalence-based approach and sometimes compromises must be made. Most studies have followed an incidence-based approach (n=25, Table 2). For some of these countries, not all incidence-based data could be gathered, and partly the prevalence-based method was used.

The GBD developed a list of disease and injury causes based on the ICD. Including all causes avoids the problems of overinclusiveness of single-cause studies and incompatible mortality claims for different causes. Most studies used the GBD disease and injury causes, and sometimes some causes were removed due to little relevance and other causes were added to the list (Table 2). Three studies developed their own disease and injury groups [20-22]. The original GBD study applied age-weighting and discounting [23]. With age-weighting, the altering levels of dependency with age are taken into account, meaning that years lived at youngest and oldest age are given less weight. Discounting means that future life years are assigned less value than those lived today. This is based on the economic concept that immediate profits are generally preferred over benefits later in time [1]. Both age-weighting and discounting have been disputed, which is further described in the discussion section. This debate is also translated in the distinction in the use of both methodologies in the included studies. Almost all studies (n=26) assigned less value to future life years by using a discount factor. However, only half of the studies (n=17) performed age-weighting in their study. It was not always stated whether age-weighting and discounting were used [18,24].

#### Methods to calculate YLL

Country-specific or model life tables with life expectancy data can be used to calculate YLLs. Most studies used the Coale and Demeny West Level 26 and 25 life tables, developed by GBD (n=23; Table 2). The West Level 26 and 25 life tables are global model life tables that have a standard life expectancy at birth: 80.0 years for males and 82.5 years for females [25]. Other studies used life tables from their country (n=8) [18,21,26-31].

#### Methods to calculate years lost due to disability (YLD)

A crucial aspect to calculating YLD is the disability weight; a value ranging from 1, indicating worst imaginable health state equal to death, through 0, indicating full health. Its value is based on the preferences stated by a panel of judges towards a set of hypothetical health states, expressing the relative undesirability of the health state [32,33]. Several sets of disability weights exist, such as the GBD disability weights [1] and the Dutch Disability Weights (DDW) [34]. Most studies (n=27; Table 2) used the GBD disability weights. Sixteen of these studies combined the GBD weights with the DDW for disease and injury causes that were not in the GBD study.

Four studies [14,29,35,36] derived YLD by applying the ratio of YLD to YLL from one study to derive the YLDs for their own study, which is common in burden of disease analyses for countries with limited data on disease occurrence [25]. For instance, the Western Australian Burden of Disease study [29] used this method to derive the YLDs for residual conditions not specifically analysed, but which were grouped to complete a broad disease grouping (e.g., other cardiovascular conditions).

Eight studies adjusted for comorbidity [12,13,16,19,20,28,30,31]. The Australian burden of disease studies [13,19,28,30] have developed methods to address the issue of comorbidity for the common coexisting nonfatal conditions (e.g., deafness, osteoarthritis, mental retardation, diabetes). With this method, the difference between a composite weight for two coexisting conditions and the weight for the more severe of the conditions is calculated and used, rather than the weight of the milder condition in its independent state. The disability weight for the more severe condition remains unchanged.

### Which methods were used to handle uncertainty and risk factor analysis?

#### Uncertainty analysis

Each burden of disease study contains uncertainty as a result of possible imprecision in epidemiological data (e.g., deaths, incidence, prevalence, severity), in the parameter values used or due to methodological controversy. None of the studies quantify uncertainty in epidemiological data. Uncertainty in the parameter values is described by sensitivity analysis in 21 studies (Table 2). These studies test whether plausible changes in values of the main variables affect the results of the analysis [1]. Most studies showed how the results of their study varied when a discount rate changes, and some studies also examined the influence of the use of age-weighting, the effect of uncertainty in the disability weights, and/or uncertainty in the incidence data.

#### Risk factor analysis

A risk factor is an attribute or exposure that is causally associated with an increased probability of a disease or injury [1]. Regarding the causal attribution of the burden of disease, one can either attribute it to a single cause (categorical attribution) or to a group of causes (counterfactual attribution). The latter can be analyzed using counterfactual analysis. With counterfactual analysis, the current or future disease burden is compared with the burden of disease that would be expected under an alternative hypothetical scenario, the counterfactual scenario, to estimate the effects of disease(s) or risk factor(s) [37].

Twelve studies performed risk factor analyses [8-13,15,19,20,26,28,29]. Risk factors that were analyzed were related to the effect of lifestyle factors (such as tobacco smoking, physical inactivity, alcohol consumption, diet, unsafe sex, and intimate partner violence), physiological states (such as obesity, high blood pressure, and high cholesterol) and also societal conditions (such as occupational exposures and air pollution) on the burden of disease.

### Comparison of disability-adjusted life year outcomes for specific disease and injury groups

The total burden due to diseases and injuries varies enormously between the included studies. The highest burden of disease was found in Pakistan (45,600 DALYs per 100,000) and Zimbabwe (41,900 DALYs per 100,000). The lowest burden of disease was estimated in Queensland (10,700 DALYs per 100,000) and Singapore (10,400 DALYs per 100,000) (Table 3). The differences in total DALYs between countries can partly be explained by differences in exposure to risk factors. For example, almost half of the total burden of disease in Zimbabwe is due to HIV and diarrhea. These diseases are rare in developed countries. In most developed countries the highest burden is caused by cardiovascular diseases, followed by road traffic injuries and depression.

**Table 3:** DALY outcomes for specific disease and injury groups (per 100,000 persons)

**Author (ref no.)**	**Country**	GBD study all causes*	**Study outcome**					
			**All causes**	**Cardiovascular diseases**	**Road traffic injury**	**Diabetes Mellitus**	**Depression**	**HIV/AIDS**
Begg [13]	England	Australia	9,894	13,240	2,380	930	720	1,760
Bowie [22]	England	11,012	n.a.	n.a.	n.a.	n.a.	n.a.	n.a.
Bradshaw [14]	South Africa	46,137	36,100	n.a.	2,800 (all unintentional)	n.a.	n.a.	10,210
Bundhamchareon [16]	Thailand	20,216	13,710	380	893	624	342	1,900
Chapman [41]	Zimbabwe	82,801	41,930	293	461	n.a.	2,350	20,380
Dodhia [50]	England	11,012	13,400	2,300	n.a.	n.a.	3,220	n.a.
Hyder [40]	Pakistan	26,693	45,630	2,420	n.a.	1,170	n.a.	109
Innove Solutions [51]	England	11,012	n.a.	n.a.	n.a.	n.a.	n.a.	n.a.
Jankovic [52]	Serbia /Serbia Montenegro	14,562	n.a.	1,800	1,800	3,000	8,800	100
Laaser [38]	Syria	16,167	14,700	3,700	1,120	610	840	n.a.
Lai [27]	Estonia	16,212	32,700	5,700	n.a.	n.a.	n.a.	n.a.
Lopez [9]	Global		Vary by region	n.a.	n.a.	n.a.	n.a.	n.a.
Mathers [25]	Australia	9,894	13,700	2,400	300	410	510	n.a.
Melse [21]	Netherlands	9,948	16,000	2,720	460	540	710	100
Michaud [35]	USA	12,844	12,420	1,730	520	n.a.	n.a.	360
Murray [10]	Global		Vary by region	n.a.	n.a.	n.a.	n.a.	n.a.
Naghavi [31]	Iran	19,432	21,570	1,840	1,960	n.a.	900	n.a.
Phua [39]	Singapore	10,111	10,400	2,050	360 (all unintentional)	1,130	1,200	n.a.
Pike [30]	Queensland	9,894	10,710	2,260	240	320	1,400	8
								
SA Dep Health, 2005 [12]	Australia	9,894	13,220	2,700	950	400	1,800	n.a.
Somerford [29]	Australia	9,894	n.a.	n.a.	n.a.	n.a.	n.a.	n.a.
Stevens [26]	Mexico	9,894	14,501	450	700	510	900	n.a.
Tobias [20]	New Zealand	10,642	15,100	3,620	450	574	1,810	n.a.
Ünüvar [15]	Turkey	16,307	14,790	2,070	355	280	590	n.a.
Victorian BOD [19]	Australia	9,894	13,600	860	230	1,120	860	25
WHO, 2008 [8]	Global		Vary by region	n.a.	n.a.	n.a.	n.a.	n.a.
WHO, 2009 [11]	Global		Vary by region	n.a.	n.a.	n.a.	n.a.	n.a.
Yoon [24]	Korea	22,128	n.a.	1,490	n.a.	990	1,140	n.a.
Yusoff [17]	Malaysia	16,638	n.a.	n.a.	n.a.	n.a.	n.a.	n.a.
Zhao [18]	Australia (non-aboriginals)	9,894	18,320	2,600	2,580 (all unintentional)	440	3,060	n.a.
Zhou, 2011 [36]	China	15,750	12,270	1,200	1,470 (all unintentional)	90	n.a.	n.a

* age-standardized DALY per 100,000 by country. Source: Global Burden of Disease study 2004. http://www.who.int/healthinfo/global_burden_disease/estimates_country/en/index.html.

Comparison with the GBD DALY outcomes by country revealed that DALY estimates were of similar magnitude in some studies (e.g., for Syria [38], USA [35], Singapore [39], and Turkey [15]). Other studies reported DALY estimates that were two times higher [27,40] or two times lower [41] than the GBD study (Table 3).

Notably, four of the Australian burden of disease studies found comparable total DALY outcomes (between 13,220 and 13,700) [12,13,19,28], where the GBD reported 9,894 as total DALYs for Australia.

## Discussion

We systematically reviewed 31 general burden of disease studies using the DALY approach and performed a quality assessment of the methodology used. We found that studies generally followed the GBD approach, but that large differences exist in methodology. Most studies used the incidence-based approach (80%), and almost all studies classified disease and injury groups as defined by the GBD. Half of the studies used age-weighting, whereas 80% of the studies used discounting.

As all systematic reviews, our study has some limitations. Reviewing the literature in the field of “burden of disease” studies was complicated by a wide variety of terminology for burden of disease. Consequently, some relevant publications may have been missed. To enhance the identification of relevant burden of disease studies we have used a variety of literature databases and keywords were matched to database-specific indexing terms. Furthermore, this review is limited to the English language. Therefore, relevant studies in other languages (e.g., Spanish [42,43]) are excluded. In the databases that were reviewed, we found a limited number of studies that included all diseases and injuries. An explanation for this finding may be that the use of the DALY is controversial and accompanied by theoretical concerns [44]. Practical concerns, such as lack of resources and available data sources and/or expertise, may also be a reason for researchers’ apprehension to perform multiple-cause burden of disease studies.

### Quality of the data and were there any data gaps

The main issue in burden of disease studies is access to complete, consistent, and comparable epidemiological data. Summary measures of population health, such as the DALY, are only as good as the weakest link in the chain, which is the epidemiological evidence [45]. Most studies derived numbers of incident cases directly from disease registers, routine databases, or epidemiological studies. Furthermore, some studies used a combination of incidence and prevalence-based data because, for most conditions, only prevalence data were available. The calculation of mortality burden is straightforward, and the precision of the estimates of YLL depends almost entirely on the quality of data on underlying causes of death. Although great improvements in reporting, coding, and classification of mortality have been made, significant challenges remain. The infrastructure for mortality and health databases varies considerably around the world [1,46]. Developed regions have electronic databases that provide summary statistics through World Wide Web-based queries. Other countries maintain tabulated mortality statistics that are not integrated into a utilizable database, and many developing countries have paper-based systems with rates based on projections and estimates rather than actual counts [46]. The data challenges that result from disparities in the level of health infrastructure yield rates that can be difficult to compare. Furthermore, differences in death certification systems, methods of data collection, and definitions of variables severely challenge international comparisons.

### Methodological choices

The calculation of the morbidity component of the burden of disease, expressed in YLD, requires extensive epidemiological modelling and is often based on a diverse range of data sources, literature research, and/or expert opinion. The resulting YLD estimates depend highly on the specific model being applied and the type of data underlying this model.

Most studies used the GBD 1996 disability weights, in many cases supplemented by DDW. The GBD disability weights cover a wider range of conditions than covered by the DDWs, but are generally less specific in terms of the disease and sequelae categories to which they refer. The set of DDW covers a more restricted range of conditions compared to the GBD 1996 disability weights, but it differentiates more finely between condition stages and severities, thus allowing more detailed disease models in estimating the YLD than is possible with the GBD weights [47]. For example, DDWs are often used for HIV/AIDS and organ disorders. For the GBD 2010 Study, new disability weights are being derived [6].

In the original GBD study, discounting and age-weighting are applied. Both age-weighting and discounting have been disputed. The use of age-weighting is discussed, since lost years of healthy life are assumed to be of equal value regardless of the age at loss, the absence of empirical foundation and validation, and because the age weights do not convey actual social values as this practice is controversial [3,48]. Discounting has been disputed because its application results in a lower efficiency of prevention programs, whereas not discounting, or the use of a low discount rate – lower than the discount rate used for the costs – favors preventive measures due to benefit in the far future [32]. This discussion is reflected in different choices to use discounting and age-weighting between studies.

To measure the gap between actual population health and an ideal, most studies used the global standard life expectancy (West Level 26 and 25 life tables) as used by the GBD study. These life tables contain the relevant expectancies for each age and sex grouping. However, they have the disadvantage that they are abridged period life tables, chiefly using five-year age groupings and an upper-age category of 85+ [28]. The use of cohort life expectancies with more complete underlying population data and more complex methods, as done for two Australian studies [12,28], resulted in more accurate and slightly different life expectancy measures. The use of the global standard life expectancy figures is recommended to enlarge the comparability with GBD study outcomes, but a sensitivity analysis using more detailed country-specific life expectancies is recommended.

### Comparison of DALY outcomes

The sensitivity of DALYs, defined by the relative contributions of “true” and “error” variation, is assumed to be low. Potential sources of true variation include differences in the size and structure of populations, real differences in disease epidemiology between populations or over time, and differences in disability weights. Error variation may originate from the use of different methodologies (e.g., for discounting and age-weighting, disability weights) and from low-quality mortality and morbidity data. The detection of true variation is the focus of interest when estimating the burden of disease in DALYs. However, error may limit the power to detect true differences between populations [49]. Therefore, at the moment burden of disease studies are not comparable, nor are disease rankings as these are affected by methodological variation as well.

## Conclusions and recommendations

Burden of disease analyses provide a unique perspective on health, one that integrates fatal and nonfatal outcomes, yet also allows the two classes of outcomes to be examined separately. Furthermore, burden of disease studies may provide a valuable insight into the scope for further health gains on the global or country level. This information will assist in taking up the future challenges posed by an aging population, by changes in disease and risk factor patterns, and by the increasing costs of health services [28]. Linking burden of disease analyses to cost effectiveness studies of interventions for major health problems will allow these interventions to be judged both in terms of cost effectiveness, and their relative impacts in reducing the burden of disease and ill health at the population level. Furthermore, burden of disease studies may shed light on crucial data gaps and facilitate priority setting in research.

However, large differences in used methodology exist between general burden of disease studies. Because of the methodological variation between studies it is difficult to assess whether differences in DALY estimates between the studies are due to actual differences in population health or whether these are the result of methodological choices. Overcoming this methodological rigor between burden of disease studies using the DALY approach is a critical priority for advancing burden of disease studies. Harmonization of the methodology used and high-quality data can enlarge the detection of true variation in DALY outcomes between populations or over time.

Furthermore, overcoming this limitation in methodological rigor is particularly important in view of the imminent launch of the GBD 2010 Study, which is expected to result in a new impulse for the performance of burden of disease studies. It is a challenge for the GBD to develop more detailed harmonization procedures and clear guidelines to increase methodological improvements and enhanced comparability of general burden of disease studies.

## Competing interest

The authors declare that they have no competing interest.

## Authors’ contributions

SP and JH carried out the search, selected included papers, independently critically appraised the selected papers, and developed the evidence tables. SP wrote the initial draft of the paper. AH was reviewer for included critically appraised papers, and contributed to the writing of the paper. JH, CS, and AH contributed substantially to the interpretation of study findings and writing the paper. All authors read and approved the final manuscript.

## References

[B1] Murray CJL Lopez AD The global burden of disease: A comprehensive assessment of mortality and disability from diseases, injuries and risk factors in 1990 and projected to 2020 1996 Cambridge: Harvard University Press

[B2] Worldbank World Development Report 1993: Investing in Health 1993 New York: Oxford University Press

[B3] Anand S Hanson K Disability-adjusted life years: a critical review J Health Econ 1997 16 685 702 10.1016/S0167-6296(97)00005-2 10176779

[B4] Barendregt JJ Bonneux L Van der Maas PJ DALYs: The age-weights on balance Bull World Health Organ 1996 74 439 443 8823967 PMC2486889

[B5] Global Burden of Disease (GBD) 2010 study http://www.who.int/healthinfo/global_burden_disease/GBD_2005_study/en/index.html

[B6] Salomon JA New disability weights for the global burden of disease Bull World Health Organ 2010 88 879 10.2471/BLT.10.084301 21124707 PMC2995197

[B7] Wielders CC van Lier EA Van't Klooster TM van Gageldonk-Lafeber AB van den Wijngaard CC Haagsma JA Donker GA Meijer A van der Hoek W Lugnér AK The burden of 2009 pandemic influenza A(H1N1) in the Netherlands Eur J Public Health 2010 in press 10.1093/eurpub/ckq187 21183472

[B8] The global burden of disease: 2004 update 2008 Geneva: World Health Organization

[B9] Lopez AD Mathers CD Ezzati M Jamison DT Murray CJ Global and regional burden of disease and risk factors, 2001: systematic analysis of population health data Lancet 2006 367 1747 1757 10.1016/S0140-6736(06)68770-9 16731270

[B10] Murray CJ Lopez AD Alternative projections of mortality and disability by cause 1990–2020: global burden of disease study Lancet 1997 349 1498 1504 10.1016/S0140-6736(96)07492-2 9167458

[B11] Global health risks: mortality and burden of disease attributable to selected major risks 2009 Geneva: World Health Organization

[B12] SE Department of Health (DoH) Population health in South Australia: burden of disease and injury estimates, 1999–2001 2005 Adelaide: Department of Health

[B13] Begg SJ Vos T Barker B Stanley L Lopez AD Burden of disease and injury in Australia in the new millennium: measuring health loss from diseases, injuries and risk factors Med J Aust 2008 188 36 40 18205562 10.5694/j.1326-5377.2008.tb01503.x

[B14] Bradshaw D Groenewald P Laubscher R Nannan N Nojilana B Norman R Pieterse D Schneider M Initial Burden of Disease Estimates for South Africa, 2000 2003 Cape Town: South African Medical Research Council 14635557

[B15] Ünüvar N Mollahaliloglu S Yardim N Turkey Burden of Disease Study 2004 Ankara: Ministry of Health

[B16] Bundhamchareon K Teerawattananon Y Vos T Begg S Burden of disease and injuries in Thailand 2002 Nonthaburi: Ministry of Public Health

[B17] Yusoff AF Mustafa AN Kaur GK Omar MA Vos T Rao VPC Begg S Malaysian Burden of Disease and Injury Study Forum 9 2005 Mumbai, India 1 24

[B18] Zhao Y Guthridge S Magnus A Vos T Burden of disease and injury in Aboriginal and non-Aboriginal populations in the Northern Territory Med J Aust 2004 180 498 502 15139825 10.5694/j.1326-5377.2004.tb06051.x

[B19] Victorian Burden of Disease Study Mortality and morbidity in 2001 2005 Melbourne: Victorian Government Department of Human Services

[B20] Tobias M The Burden of Disease and Injury in New Zealand 2001 Wellington: Ministry of Health

[B21] Melse JM Essink-Bot ML Kramers PG Hoeymans N A national burden of disease calculation: Dutch disability-adjusted life-years. Dutch burden of disease group Am J Public Health 2000 90 1241 1247 10937004 10.2105/ajph.90.8.1241 PMC1446331

[B22] Bowie C Beck S Bevan G Raftery J Silverton F Stevens A Estimating the burden of disease in an English region J Public Health Med 1997 19 87 92 10.1093/oxfordjournals.pubmed.a024595 9138224

[B23] Murray CJ Quantifying the burden of disease: the technical basis for disability-adjusted life years Bull World Health Organ 1994 72 429 445 8062401 PMC2486718

[B24] Yoon SJ Bae SC Lee SI Chang H Jo HS Sung JH Park JH Lee JY Shin Y Measuring the burden of disease in Korea J Korean Med Sci 2007 22 518 523 10.3346/jkms.2007.22.3.518 17596664 PMC2693648

[B25] Mathers CD Vos T Lopez AD Salomon JA Ezzati M National Burden of Diseases Studies: A Practical Guide. Edition 2.0 2001 Geneva: WHO Global Program on Evidence for Health Policy

[B26] Stevens G Dias RH Thomas KJ Rivera JA Carvalho N Barquera S Hill K Ezzati M Characterizing the epidemiological transition in Mexico: national and subnational burden of diseases, injuries, and risk factors PLoS Med 2008 5 e125 10.1371/journal.pmed.0050125 18563960 PMC2429945

[B27] Lai T Habicht J Kiivet RA Measuring burden of disease in Estonia to support public health policy Eur J Public Health 2009 19 541 547 10.1093/eurpub/ckp038 19401358

[B28] Mathers CD Vos ET Stevenson CE Begg SJ The burden of disease and injury in Australia Bull World Health Organ 2001 79 1076 1084 11731817 PMC2566696

[B29] Somerford P Katzenellenbogen J Western Australian Burden of Disease Study: Disability-. Adjusted Life Years: Technical overview 2004 Perth: Department of Health

[B30] Pike A Baade P Harper C Muller S Kennedy B Quantifying the burden of disease and injury in Queensland 1996–1998 2002 Brisbane, Australia: Queensland Government

[B31] Naghavi M Abolhassani F Pourmalek F Lakeh M Jafari N Vaseghi S Mahdavi Hezaveh N Kazemeini H The burden of disease and injury in Iran 2003 Popul Health Metr 2009 7 9 10.1186/1478-7954-7-9 19527516 PMC2711041

[B32] Drummond M O'Brien B Stoddart GL Torrance GW Methods for the Economic Evaluation of Health Care Programmes 1997 Oxford: Oxford Medical Publications

[B33] Murray CJ Acharya AK Understanding DALYs (disability-adjusted life years) J Health Econ 1997 16 703 730 10.1016/S0167-6296(97)00004-0 10176780

[B34] Stouthard MEA Essink-Bot ML Bonsel GJ DUTCH DISABILITY WEIGHTS (DDW) GROUP Disability weights for diseases; a modified protocol and results for a Western European Region Eur J Public Health 2000 10 24 30 10.1093/eurpub/10.1.24

[B35] Michaud CM McKenna MT Begg S Tomijima N Majmudar M Bulzacchelli MT Ebrahim S Ezzati M Salomon JA Kreiser JG The burden of disease and injury in the United States 1996 Popul Health Metr 2006 4 11 10.1186/1478-7954-4-11 17049081 PMC1635736

[B36] Zhou SC Cai L Wang J Cui SG Chai Y Liu B Wan CH Measuring the burden of disease using disability-adjusted life years in Shilin County of Yunnan Province, China Environ Health Prev Med 2011 16 148 154 10.1007/s12199-010-0176-8 21431803 PMC3078293

[B37] Maldonado G Greenland S Estimating causal effects Int J Epidemiol 2002 31 422 429 10.1093/ije/31.2.422 11980807

[B38] Laaser U The Burden of Diseases and Injury in Syria 2007 Damascus: Ministry of Health

[B39] Phua HP Chua AV Ma S Heng D Chew SK Singapore's burden of disease and injury 2004 Singapore Med J 2009 50 468 478 19495514

[B40] Hyder AA Morrow RH Applying burden of disease methods in developing countries: a case study from Pakistan Am J Public Health 2000 90 1235 1240 10937003 10.2105/ajph.90.8.1235 PMC1446325

[B41] Chapman G Hansen KS Jelsma J Ndhlovu C Piotti B Byskov J Vos T The burden of disease in Zimbabwe in 1997 as measured by disability-adjusted life years lost Trop Med Int Health 2006 11 660 671 10.1111/j.1365-3156.2006.01601.x 16640619

[B42] Gènova-Maleras R Álvarez-Martín E Catalá-López F Fernández de Larrea-Baz N M-G C Burden of disease in the elderly population in Spain Gac Sanit 2011 25 47 50 Spanish 22138281 10.1016/j.gaceta.2011.09.018

[B43] Gómez Dantés HCM Franco-Marina F Bedregal P Rodríguez García J Espinoza A Valdez Huarcaya W América. LRRdIsCdEdOdSIp Burden of disease in Latin America [Article in Spanish] Salud Publica Mex 2011 53 72 77 21877095

[B44] Gouda HN Powles JW Why my disease is important: metrics of disease occurrence used in the introductory sections of papers in three leading general medical journals in 1993 and 2003 Popul Health Metr 2011 9 14 10.1186/1478-7954-9-14 21605431 PMC3118323

[B45] Polinder S Haagsma JA Lyons RA Gabbe BJ Ameratunga S Cryer C Derrett S Harrison JE Segui-Gomez M van Beeck EF Measuring the population burden of fatal and nonfatal injury Epidemiol Rev 2012 34 17 31 10.1093/epirev/mxr022 22113244

[B46] Chandran A Hyder AA Peek-Asa C The global burden of unintentional injuries and an agenda for progress Epidemiol Rev 2010 32 110 120 10.1093/epirev/mxq009 20570956 PMC2912603

[B47] Essink-Bot ML Bonsel GJ Murray CJM, Salomon JA How to derive disability weights Summary measures of population health: concepts, ethics, measurement and applications 2002 Geneva: World Health Organization

[B48] Johanneson M Johansson PO Is the valuation of QALY's gained independent of age? some empirical evidence J Health Econ 1997 16 589 599 10.1016/S0167-6296(96)00516-4 10175633

[B49] Essink-Bot ML Pereira J Packer C Schwarzinger M Burstrom K Cross-national comparability of burden of disease estimates: the European disability weights project Bull World Health Organ 2002 80 644 652 12219156 PMC2567594

[B50] Dodhia H Phillips K Measuring burden of disease in two inner London boroughs using disability adjusted life years J Public Health (Oxf) 2008 30 313 321 10.1093/pubmed/fdn015 18400697

[B51] Centre for Health Care Development, West Pennine Health Authority and Living Media Ltd West Pennine Burden of Disease Report 1998

[B52] Jankovic S Vlajinac H Bjegovic V Marinkovic J Sipetic-Grujicic S Markovic-Denic L Kocev N Santric-Milicevic M Terzic-Supic Z Maksimovic N Laaser U The burden of disease and injury in Serbia Eur J Public Health 2007 17 80 85 10.1093/eurpub/ckl072 16751634

[B53] Stouthard ME Essink-Bot ML Bonsel GJ Disability weights for diseases. A modified protocol and results for a Western European region Eur J Public Health 2000 10 24 30 10.1093/eurpub/10.1.24

